# The Effect of Smartphone App-Use Patterns on the Performance of Professional Golfers

**DOI:** 10.3389/fpsyg.2021.678691

**Published:** 2021-05-24

**Authors:** Jea Woog Lee, Jae Jun Nam, Kyung Doo Kang, Doug Hyun Han

**Affiliations:** ^1^Department of Sport Technology and Information, Chung-Ang University, Anseong, South Korea; ^2^Department of Golf, Korea Golf University, Hoeng Seong, South Korea; ^3^Department of Psychiatry, Chung-Ang University Hospital, Seoul, South Korea

**Keywords:** golf, smartphone, applications, performance, handicap

## Abstract

Smartphone app-use patterns will predict professional golfers’ athletic performance, and the use time of serious apps would be associated with improved performance. This longitudinal 4-week observation of 79 professional golfers assessed golf handicaps and smartphone app-use patterns at the start of the Korean professional golf season and 2 and 4 weeks later. We classified use as social networking, entertainment, serious apps, and others. Use time of entertainment apps increased for non-improved golfers but did not change for improved golfers. Use time of serious apps increased for improved golfers and decreased for non-improved ones. Changes in golf handicaps were positively correlated with changes in entertainment app use time and negatively correlated with changes in serious app use time. Professional golfers’ sports performance was not associated with smartphone use time but was with the smartphone app type. The management of smartphone app-use patterns is important for professional golfers’ performance.

## Introduction

In recent decades, smartphones based on mobile networks have become indispensable in the regular lives of people, and the rate of their usage has continuously increased (Silver, 2019). In a survey of smartphone ownerships and internet usage rates in 39 countries, 75% of the participants used either the internet or their own smartphones (Joe and Knight, 2019). Among advanced economies, South Korea has the highest smartphone ownership rate of 96% (Kim et al., 2012; Silver, 2019). In 2011, smartphone ownership in Korea was 38.3%, ranking fourth in the world (Poushter et al., 2018). Over the past 10 years, the ownership of smartphones in Korea has rapidly increased, and smartphone usage very closely affects the general life of Koreans (Winskel et al., 2019). In the United States, smartphone ownership also rapidly increased, from 37% in 2001 to 92% in 2019 (Silver, 2019).

With the rapid increase in smartphone ownership and usage, the number and popularity of mobile applications (apps) has also increased tremendously. There were 150,000 apps and 350,000 activations in the Android market, as well as 350,000 apps and 10 billion activations in App Store on iOS (Schmidt, 2011). In an analysis of smartphone apps, Kim et al. (2014) suggested the types of apps as follows: book, business, education, entertainment, finance, games, healthcare, lifestyle, medical, music, navigation, news, photography, productivity, reference, social network service (SNS), sports, travel, utilities, and weather. In addition, diverse uses of smartphone apps have been associated with geographic locations, similarity of contents, and interest of users (Xu et al., 2011).

Typically, people are motivated to use mass media and the internet to satisfy social support needs, including companionship, to pass time, out of habit, and escape, as well as for mood management, including relaxation, entertainment, arousal, and information (Papacharissi and Rubin, 2000; Aderibigbe and Armide, 2012). Recently, smartphone apps have been applied to health promotion, education, and human behavioral changes (Kratzke and Cox, 2012).

The sport of golf is uniquely challenging, because its duration, interrupted pace of play, and excessive idle time make the competitor vulnerable to external distractors (Singer, 2002), which can result from things in an athlete’s physical environment, such as noise, people, television, and smartphones (Moran, 2016). The potential negative effects of smartphone apps on an athlete’s performance have been suggested in recent years (Greco et al., 2017).

Specifically, entertainment apps, such as SNS, are closely linked with athletes’ mood before competition. Some athletes have announced their decision to stop using entertainment apps during competition in order to minimize the potential for distraction (Logue, 2016). Emily Seebohm, who was one of Australia’s contenders for winning a gold medal in swimming at the London Olympics, conceded that her constant use of social media might have worsened her performance (Hayes, 2019). Several studies have reported adverse effects of smartphone use on athletes. Fortes et al. (2019) suggested that 30 min exposure to smartphone apps causes mental fatigue and impairs decision making in male soccer athletes. Park (2017) reported that excessive use of smartphones would worsen learning attitudes, relationships with others, and self-control in college golf players.

On the other hand, smartphone use can also improve athletic performance and general life. Smartphone apps are actively used to measure neuromuscular performance, assess vital signals, prevent injury, and improve skills (Kidman et al., 2016; Driller et al., 2017; Perrotta et al., 2017; Matos et al., 2019). Using these apps may improve the competition process and the quality of training in athletes. In fact, athletes and coaches have been using various apps to improve their performance. Driller et al. (2017) measured counter-movement jumps in recreational athletes using smartphone apps. Perrotta et al. (2017) estimated the immediate heart-rate variability in athletes using smartphone apps. Matos et al. (2019) suggested that a smartphone recommendation system could prevent potential risks of injury for athletes. Kidman et al. (2016) found that wearing a device with inertial-motion tracking and vibro-tactile feedback increased the accuracy of diving movements in athletes. In a review of studies of performance support apps, Peart et al. (2019) reported various mobile apps supporting improvement of performance by measuring heart rate, range of motion, barbell velocity, vertical jump, running mechanism, and distances during walking, jogging, and running. Lim (2020) proposed an application that helps prevent injuries by analyzing the performance, fatigue, and weakness by using a camera-based mobile interface to measure the athlete’s movements. Previous research on sports psychology has suggested that behavioral patterns accompanied by self-management plans are closely related to confidence improvement (Bell, 1983). A study on athletes’ well-being and confidence provided by digital coaching in mobile applications (Kettunen et al., 2018) and on athletes’ motivation provided by applications that support athletes’ sleep monitoring and management (Halson, 2019) verified the effectiveness of self-management based on sports psychology. In video games, “serious” was prepended to refer to games used for education, scientific exploration, health care, and politics in contrast to entertainment (Lugmayr et al., 2017). As with video game, we classified apps that were used for education, scientific exploration, and health care as serious apps.

### Hypothesis and Aims

We hypothesized that smartphone app-use patterns would predict athletic performance for professional golfers. In particular, we believe that the use time of serious apps is closely associated with improved performance in professional golfers.

Our main aim in this study is to identify crucial factors for improving performance in professional golfers between smartphone use time and app-use patterns. Our secondary aim is to confirm the usefulness of serious apps for improving performance in professional golfers.

## Materials and Methods

### Participants

At the start of the Korean Professional Golfers Association (PGA) and Korean Ladies Professional Golfers Association (LPGA) season in 2020, we recruited 27 PGA golfers and 52 LPGA golfers to participate in this study. Given that we planned to compare two categories (golf handicaps and smartphone use time) between the two groups (improved group vs. non-improved group), we assessed the data of the 84 participants using GPower 3.1 software (effect size = 0.2, α error = 0.05, power = 0.95; Faul et al., 2007). The mean ± standard deviation of age, education years, and golf career years in participants (male vs. female; 27 vs. 52) were 23.8 ± 4.2 years, 11.9 ± 1.8 years, and 8.5 ± 2.7 years, respectively. There were 55 participants who had iPhones and 24 who had Android phones. The participants’ mean smartphone use time per week was 29.2 ± 10.1 h/week. We explained the goals and procedures of the study to all the golfers who agreed to participate in the research. The study was approved by the Chung Ang University Institutional Review Board (reference number: 1041078-202009-HRSB-290-01), and all participants completed and signed consent forms. We conducted this study in accordance with the principles of the Declaration of Helsinki.

### Study Design

We designed the study as a 4-week observation. At the start of the Korean professional golf season, male and female professional golfers agreed to participate in the study. They responded to questions on age, sex, years of education, golf career, golf scores, and smartphone model. The pattern of smartphone use time is described below. They were asked to provide their smartphone use time for 3 weeks. After 4 weeks, we classified all the golfers into two groups: the performance improvement group (improved golfers) and the performance non-improvement group (non-improved golfers).

The performance-improvement group was defined as the golfers who had the mean modified golf score of the total rounds at week 4 as being less than that at baseline (week 1). The baseline modified golf score was defined as the mean modified golf score of all rounds in the last year (2019). To adjust for the difficulty of the golf course, we calculated modified golf scores by adapting the World Handicap System (Driller et al., 2017). The modified golf score could be calculated as follows: subtracting the course rating from the participant’s score, multiplying the result by 113, then dividing it by the slope rating. At 4 weeks, 41 golfers showed improved golf handicap scores, from 72.2 ± 2.1 to 69.8 ± 2.0 (Improved golfers), and 38 golfers showed non-improved golf handicap scores, from 73.2 ± 4.1 to 76.2 ± 3.5 (Non-improved golfers).

### Smartphone App-Use Patterns

All the golfers voluntarily provided their smartphone use time. In the “total screen time” on the iPhone and digital “well-being use time” on Android phones, the use time of all apps for 1 week was captured and sent to the research team every Friday night (21:00/9:00 pm) for 3 weeks.

Based on use time and frequency, Xu et al. (2011) classified 3,500 apps into 20 categories such as book, business, education, entertainment, finance, games, healthcare, lifestyle, medical, music, navigation, news, photography, productivity, reference, social network service (SNS), sports, travel, utilities, and weather. Most studies of mobile apps and sports suggested that social-networking apps could affect sports competition (Smith and Sanderson, 2015; Encel et al., 2017). Considering those reports, we simplified the 20 categories into 4 app types: social networking (SNS, blog, café, face time), entertainment (YouTube, music, Netflix, pop-cast, webtoon, media apps, games), serious apps (internet browser, books and reference, creativity [camera, photo, video], education, golf form analysis apps, diary), and others (banking, shopping, delivery apps).

### Statistical Analysis

We tested the normality of all data using the Kolmogorov-Smirnov test (K-S test), calculated skewness and kurtosis, and analyzed the demographic characteristics, golf career, and smartphone use of improved and non-improved golfers using an independent *t*-test or chi-squared test. We assessed the effect size of independent *t*-tests with Cohen’s d, which we interpreted as follows: 0.0 < *d* < 0.2, small; 0.3 < *d* < 0.5, medium; *d* > 0.6, large (Cohen, 1988). We analyzed sex distribution between the two groups using a chi-squared test. We assessed the effect size of the chi-squared tests using Cramer’s V and interpreted it as follows: 0 < V < 0.5, no or very weak; 0.05 < V < 0.10, weak; 0.10 < V < 0.15, moderate; 0.15 < V < 0.25, medium; and *V* > 0.25, very strong (Cramér, 1946). We analyzed the changes in modified golf scores, as well as the total use time of smartphones and of each app of the two groups using a repeated measure ANCOVA considering age and golf career. In a *post hoc* test applied by correcting *p* < 0.05 for the number of comparisons, the significance was set at *p* < 0.0125 (0.05/4). We assessed the effect size of ANCOVA with partial eta-squared and interpreted it as follows: partial η*2* = 0.01–0.09, small; η*2* = 0.09–0.25, medium; and η*2* > 0.25, large (Bakeman, 2005). In a multiple hierarchical regression analysis of smartphone use patterns, we added a discrete set of hierarchical variables: Model 1, social network services; Model 2, social network services + entertainment apps; Model 3, social network services + entertainment apps + serious apps; and Model 4: social network services + entertainment apps + serious apps + other apps. The dependent variable of “improved golfers” was coded as “1” and “non-improved golfers” was coded as “0.” As mentioned above, we defined the improved golfers as golfers whose modified golf scores of total rounds at week 4 were less than those at the baseline (week 1).

Hierarchical regression analysis can show a significant amount of variance in the dependent variable considering all other variables. We verified the overall fit of each step of the logistic regression model with χ2-values (model χ2 and step χ2) as well as goodness-of-fit indices represented with “-2 log likelihood.” The χ2 values showed the improvement observed in the model, with the predictors relative to the constant-only model or the model preceding the current model. To evaluate the practical usefulness of each model, we also used tables of classification accuracy to assess the relative success of each model in predicting the correlations with improved golfers. In addition to the indices of the overall model fit, we assessed Nagelkerke’s R2 as an approximate estimate of how variance in the dependent variable was accounted for by the model. To test whether each individual factor had a significant relationship with improved golfers, we used Wald statistics. When a significant relationship was detected by the Wald test, we interpreted the coefficient by finding the odds ratio, that is, the ratio between the probability that the event (i.e., improved golfers) would occur and the probability that it would not.

## Results

### Testing for Normality of Data

None of the data differed significantly from the normal distribution. These included:

• age (Improved golfers: K-S test statistic (D) = 0.13, *p* = 0.48, skewness *z* = −0.01, kurtosis *z* = −0.18.
• Non-improved golfers: *D* = 0.18, *p* = 0.17, skewness *z* = 1.82, kurtosis *z* = 1.69).
• golf career length (Improved golfers: *D* = 0.18, *p* = 0.14, skewness *z* = 0.44, kurtosis *z* = 0.84.
• Non-improved golfers: *D* = 0.15, *p* = 0.34, skewness *z* = 0.85, kurtosis *z* = 0.80).
• golf scores at baseline (Improved golfers: *D* = 0.15, *p* = 0.32, skewness *z* = 0.35, kurtosis *z* = 1.385.
• Non-improved golfers: *D* = 0.20, *p* = 0.07, skewness *z* = 0.20, kurtosis *z* = −1.16), and
• smartphone use time (Improved golfers: *D* = 0.13, *p* = 0.44, skewness *z* = 0.56, kurtosis *z* = −0.24.
• Non-improved golfers: *D* = 0.11, *p* = 0.70, skewness *z* = 0.52, kurtosis *z* = −0.19).

### The Comparison of Demographic Data and the Changes in Handicaps Between Improved and Non-improved Golfers

**FIGURE 1:**
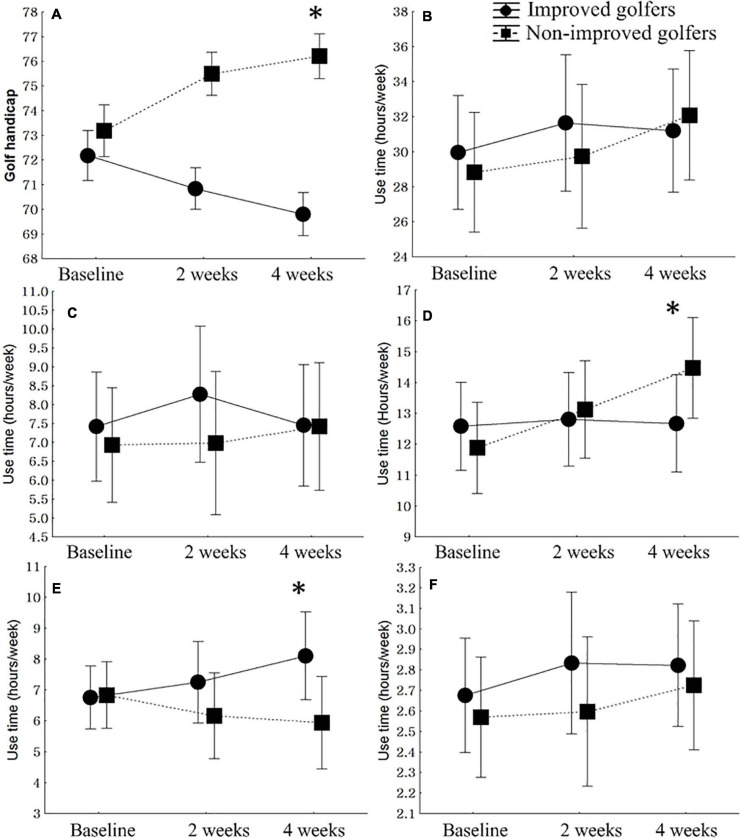
Comparison of changes in modified golf scores and smartphone use time of improved and non-improved golfers. **(A)** Golf handicap, *F* = 57.76, *p* < 0.01, η*2* = 0.4513. **(B)** Total smartphone use time, *F* = 2.84, *p* = 0.09, η*2* = 0.123. **(C)** Social network service, *F* = 2.69, *p* = 0.08, η*2* = 0.262. **(D)** Entertainment apps (media applications including listening to music and watching movies and dramas, webtoons, games), *F* = 5.03, *p* = 0.01, *2*η = 0.308. **(E)** Serious apps (internet browsers, books and reference apps, camera, swing analysis apps, schedule apps, education), *F* = 5.25, *p* < 0.01, η*2* = 0.353. **(F)** Other apps (shopping, delivery service, etc.), *F* = 1.11, *p* = 0.30, η*2* = 0.007.

There were no significant differences in age, sex distribution, education year, golf career, or modified golf score between the improved and non-improved golfers (Table 1). Over the course of 4 weeks, the modified golf scores of the improved golfers decreased more than those of the non-improved golfers (*F* = 57.76, *p* < 0.01) (Figure 1).

**TABLE 1:** Demographic data and smartphone use time.

**Variables**	**Improved golfers (*n* = 41)**	**Non-improved golfers (*n* = 38)**	**Statistics**
Age (years)	23.5 ± 3.3	24.0 ± 5.0	*t* = −0.55, *p* = 0.58, ES = 0.12
Sex (male/female)	13/28	14/24	χ^2^ = 0.23, *p* = 0.63, ES = 0.01
Education years	11.5 ± 1.7	12.3 ± 1.7	*t* = −1.87, *p* = 0.06, ES = 0.47
Golf career (years)	8.8 ± 2.6	8.3 ± 2.9	*t* = 0.87, *p* = 0.38, ES = 0.18
Golf scores at baseline	72.1 ± 2.2	73.2 ± 4.1	*t* = −1.49, *p* = 0.13, ES = 0.33
Smartphone model (iOS/Android)	32/9	23/15	χ^2^ = 2.86, *p* = 0.09, ES = 0.01
Smartphone use time (hours/week)	29.9 ± 10.7	28.8 ± 10.2	*t* = 0.48, *p* = 0.63, ES = 0.11
SNS	7.4 ± 3.9	6.9 ± 5.3	*t* = 0.46, *p* = 0.64, ES = 0.11
Entertainment	12.6 ± 5.3	11.9 ± 3.7	*t* = 0.62, *p* = 0.53, ES = 0.15
Serious apps	6.8 ± 3.4	6.8 ± 3.0	*t* = −0.10, *p* = 0.91, ES < 0.01
Other apps	2.7 ± 1.0	2.6 ± 0.8	*t* = 0.53, *p* = 0.60, ES = 0.11

*SNS, social network service; apps, applications; ES, effect size.*

During the 4-week period, there were no significant differences in total smartphone use time between improved and non-improved golfers (*F* = 2.84, *p* = 0.09, η*2* = 0.123). There were no significant differences in the time spent using SNS (*F* = 2.69, *p* = 0.08, η*2* = 0.262) or other apps (*F* = 1.11, *p* = 0.30) between improved and non-improved golfers. However, there were significantly different changes in the use time of entertainment and serious apps between improved and non-improved golfers. During the 4-week period, the use time of entertainment apps of non-improved golfers increased, although that of improved golfers did not change (*F* = 5.03, *p* = 0.01). Furthermore, improved golfers’ use time for serious apps increased, whereas that of non-improved golfers decreased (*F* = 5.25, *p* < 0.01) (Figure 1).

### The Comparison of Smartphone Use Time and Smartphone Use Patterns Between Improved and Non-improved Golfers

There were no significant differences between the two groups in the smartphone model, total smartphone use time, SNS use time, entertainment app use time, serious app use time, or other app use time.

Of the four models we suggested, models 2, 3, and 4 were significantly associated with improved golfers. In model 2 (model 1 + entertainment apps), model χ2 (12.4, *p* = 0.03), and Nagelkerke’s R2 (0.252, 25.2% of the variance in the dependent variable of the improved golfers) indicated that the model was adequate for predicting improved golfers. With step χ2 (8.0, *p* = 0.04), entertainment apps could predict improved golfers. In model 3 (model 2 + serious apps), model χ2 (27.8, *p* < 0.01), and Nagelkerke’s R2 (0.458, 45.8% of the variance in the dependent variable of the improved golfers) indicated that the model was adequate for predicting improved golfers. With step χ2 (15.3, *p* = 0.02), serious apps could predict improved golfers. In model 4 (model 3 + other apps), model χ2 (34.7, *p* < 0.01), and Nagelkerke’s R2 (0.579, 57.9% of the variance in the dependent variable of the improved golfers) indicated that the model was adequate for predicting improved golfers. However, with step χ2 (6.9, *p* = 0.07), other apps could not predict improved golfers.

According to the Wald test for all independent variables, increased use time of serious apps at week 4 and decreased use time of entertainment apps at week 4 were significant predictors of improved golfers (Table 2).

**TABLE 2:** Hierarchical logistic regression analysis of the four models.

		**Model 1**			**Model 2**		
**App category**		**B**	**Wald**	**OR**	**B**	**Wald**	**OR**
Social network services	Baseline	0.001	0.000	1.001	0.072	0.460	1.074
	2nd week	0.196	3.365	1.216	0.128	1.434	1.137
	4th week	−0.197	2.750	0.821	−0.153	1.730	0.858
Entertainment apps	Baseline				0.073	1.265	1.076
	2nd week				0.071	0.349	1.074
	4th week				−0.207	3.854	0.829*

**Indices**	**Model 0**	**Model 1**			**Model 2**		

−2 log likelihood	107.891	103.491			95.488		
Step χ^2^/p	N/A	4.4/0.22			8.0/0.04		
Model χ^2^/p	N/A	4.4/0.22			12.4/0.03		
Nagelkerke’s R^2^	N/A	0.074			0.252		
Classification accuracy (%)	52.6	53.8			63.3		

		**Model 3**			**Model 4**		
		**B**	**Wald**	**OR**	**B**	**Wald**	**OR**

Social network services	Baseline	0.082	0.565	1.086	−0.209	1.647	0.811
	2nd week	0.084	0.607	1.088	0.079	0.392	1.082
	4th week	−0.106	0.764	0.900	−0.099	0.464	0.906
Entertainment apps	Baseline	0.171	3.711	1.187	0.175	2.951	1.370
	2nd week	0.034	0.062	1.034	0.281	2.535	1.324
	4th week	−0.203	3.872	0.817*	−0.265	4.829	0.767*
Serious apps	Baseline	−0.118	1.157	0.888	−0.132	0.976	0.877
	2nd week	−0.043	0.101	0.958	−0.080	0.303	0.923
	4th week	0.421	7.383	1.547**	0.378	7.002	1.460**
Other apps	Baseline				0.398	3.590	0.397
	2nd week				−0.976	3.538	0.377
	4th week				−0.390	1.810	0.677

**Indices**		**Model 3**			**Model 4**		

−2 log likelihood		83.174			73.236		
Step χ^2^/p		15.3/0.02			6.9/0.07		
Model χ^2^/p		27.8/< 0.01			34.7/< 0.01		
Nagelkerke’s R^2^		0.458			0.579		
Classification accuracy (%)		79.2			84.2		

OR, odds ratio. Dependent variable: Improved golfers. Model 1: social network services, Model 2: social network services + entertainment apps. Model 3: social network services + entertainment apps + serious apps. Model 4: social network services + entertainment apps + serious apps + others. *p < 0.05, **p < 0.01.

## Discussion

In this study, sports performance was not associated with smartphone use time. We found that the usage time, one of the smartphone usage patterns, could not predict the performance of a professional golfer. However, the improved golfer group showed increased use time for serious apps, whereas the non-improved golfer group showed increased use time for entertainment apps during the 4 weeks. In addition, the improvement of golf scores was correlated with serious app use time and the worsening of golf scores with entertainment app use time.

We found that the effect of SNS use on competition was not significant; SNS usage, one of the smartphone usage patterns, could not predict the performance of professional golfers. Previous studies are divided on whether the association between SNS and athletes’ performance is negative or positive. Encel et al. (2017) reported that SNS use immediately before sports competitions could increase sports anxiety. In addition, Smith and Sanderson (2015) found that players spent much time on media exposure and communication, or that their concerns about media exposure naturally led to long-term use of smartphones.

On the other hand, Hayes (2019) saw that players can receive support by communicating via SNS activities themselves. If this becomes a routine before and after the game, it can have a positive effect on their sports performance. Altogether, studies show different perspectives on the degree of helpfulness of SNS for performance, based on the individual’s disposition. In other words, it is difficult to see the use of SNS as the criterion for predicting athletes’ performance.

### Negative Correlation Between Negative Effect of Smartphone App and Performance in Professional Golfers

In this study, entertainment apps were negatively correlated with golf performance. Entertainment app usage, one of the smartphone usage patterns, is expected to worsen the performance of professional golfers. The longest smartphone game-playing time (≥3 h) was significantly associated with musculoskeletal pain in student athletes (Sanderson et al., 2020). In other words, smartphone games can increase the smartphone usage time itself and can disturb daily life, including performance itself.

In addition, Bagherianfar et al. (2017) reported that the time spent on mobile messenger software on smartphones was associated with mental-health problems, including anxiety and depression, in physical-education students. In a survey of 333 university student athletes, internet addiction was negatively correlated with group cohesion and social support (Cao and Chi, 2016). Grall-Bronnec et al. (2016) reported that internet gambling problems negatively affect training and sleep in some players. In other words, the isolation from social relationships while using the internet and the poor physical condition caused by irregularities in life rhythms seem to cause problems in the psychological and mental aspects that determine performance.

Notably, in our study, SNS did not show a significant correlation with the sports performance of professional golfers. However, long-term use of SNS by athletes has been found to worsen their performance (Billings et al., 2016; Geurin-Eagleman and Burch, 2016). In a study of male soccer athletes, 30 min of smartphone apps, including SNS, caused mental fatigue and impaired decision making (Fortes et al., 2019). It is worth looking into the types of content shared on SNS. SNS apps such as Facebook, Instagram, and Snapchat are services that help in sharing photos and videos. Athletes may spend time uploading videos and photos or viewing other people’s media content rather than communicating via text messages (Shreffler et al., 2016). In this study, it was difficult to confirm what specific activities and contents have been enjoyed from SNS, but we found that the use of smartphones using media (photos, videos, music, etc.) in the category of entertainment apps showed a significant decline in performance.

### Positive Relationship Between Positive Effect on Smartphone App and Performance in Professional Golfers

Among the smartphone application types, serious apps, which the professional golfer group mainly used, had a positive and significant correlation with performance. In contrast to the negative effects of smartphone apps on sports performance, several apps have been used to improve it. In particular, smartphone apps have been applied to control sleep time schedules and sport performance analyses (Roos, 2014; Voight et al., 2017; Balsalobre-Fernández et al., 2018; Budiono et al., 2018, 2019; Harris et al., 2018). Harris et al. (2018) reported that smartphone learning apps could improve sports performance by encouraging identity, goal awareness, positive reinforcement, and the need for convenience with limited time. Athletes who did dietary planning using the diet education app Nutriatlet showed an increase in energy consumption level ≥10% per time unit compared to athletes who did not use it (Budiono et al., 2018). In addition, Nutriatlet improved energy consumption, body mass index, and body fat percentage in martial-arts athletes (Budiono et al., 2019). In other words, the continuous use of the educational apps, which our study included in the category “Serious Apps,” leads to improvement in behavior, lifestyle, and health, thereby contributing directly and indirectly to improving performance. Moreover, athletes’ performance can be improved by means of the apps that assist in scheduling, which more conveniently support self-management and condition management, which can be difficult or overlooked by a busy player.

The effectiveness and validity of smartphone apps for sport performance analyses are similar to those of software programs on laptop computers (Balsalobre-Fernández et al., 2018). In an analysis of surveys of golfers and golf coaches, a video analysis app for golfers helped to improve overall golf performance, including stance, balance, and swing (Roos, 2014). A neuromuscular training program smartphone app improved jump-correcting lower-limb alignment during drop-jump, take-off, and landing tasks in volleyball players (Voight et al., 2017). Performance analysis apps can be highly specialized for the different components of athletes’ performance. If they are actively used, they can directly and indirectly contribute to the performance of pro golfers.

Taken together, time consumed by using entertainment apps worsened golf performance, but using serious apps increased productive behaviors in professional golfers. Based on these results, we suggest that the management and control of smartphone app-use patterns may be important for the golf performance of professional golfers. In addition, we cautiously suggest that large data and information about smartphone app-use patterns may predict golf performance.

### Limitations

This study had several limitations. First, the classification of smartphone apps was arbitrary, although we modified and applied other researchers’ classifications. In addition, we did not use formal scales with validation to assess smartphone use patterns. Second, we evaluated golf performance using golf handicaps. Finally, our having only a few subjects and only one type of sport (golf) means that our results cannot be generalized to all athletes. Future studies should include various methods, such as swing, distance, and form analyses in a large population.

## Conclusion

For professional golfers, sports performance was not associated with smartphone use time, but the type of smartphone apps was. Hence golfers’ interests, represented by smartphone apps, could predict golfers’ performance. Self-monitoring or team monitoring of the use pattern of entertainment apps and serious apps could help golfers improve their performance. Future studies should focus on the management of smartphone app use to encourage sports performance in other sports.

## Data Availability Statement

The raw data supporting the conclusions of this article will be made available by the authors, without undue reservation.

## Author Contributions

JWL, JJN, and DHH contributed to conception and design of this study. JWL and JJN collected the database. DHH produced the statistical analysis. JWL and KDK wrote sections of the manuscript and contributed to the manuscript revision, read, and approved the submitted version.

## Conflict of Interest

The authors declare that the research was conducted in the absence of any commercial or financial relationships that could be construed as a potential conflict of interest.
